# Therapeutic Use of Botulinum Toxin in Neurorehabilitation

**DOI:** 10.1155/2012/802893

**Published:** 2011-09-14

**Authors:** Domenico Intiso

**Affiliations:** Neuro-Rehabilitation Unit, Scientific Institute, Hospital IRCSS “Casa Sollievo della Sofferenza”, Viale dei Cappuccini 1, 71013 San Giovanni Rotondo (Foggia), Italy

## Abstract

The botulinum toxins (BTX), type A and type B by blocking vesicle acetylcholine release at neuro-muscular and neuro-secretory junctions can result efficacious therapeutic agents for the treatment of numerous disorders in patients requiring neuro-rehabilitative intervention. Its use for the reduction of focal spasticity following stroke, brain injury, and cerebral palsy is provided. Although the reduction of spasticity is widely demonstrated with BTX type A injection, its impact on the improvement of dexterity and functional outcome remains controversial. The use of BTX for the rehabilitation of children with obstetrical brachial plexus palsy and in treating sialorrhea which can complicate the course of some severe neurological diseases such as amyotrophic lateral sclerosis and Parkinson's disease is also addressed. Adverse events and neutralizing antibodies formation after repeated BTX injections can occur. Since impaired neurological persons can have complex disabling feature, BTX treatment should be viewed as adjunct measure to other rehabilitative strategies that are based on the individual's residual ability and competence and targeted to achieve the best functional recovery. BTX therapy has high cost and transient effect, but its benefits outweigh these disadvantages. Future studies must clarify if this agent alone or adjunctive to other rehabilitative procedures works best on functional outcome.

## 1. Introduction

Botulinum toxins are some of the most potent poisons present in nature produced by the anaerobic bacterium *Clostridium Botulinum. *Historically, these toxins were predominantly associated with a food-borne toxicosis producing a neurological life-threatening disease called “botulism”, characterized by a severe generalized muscular paralysis and cholinergic autonomic blockade. Currently, botulinum toxins have become established as efficacious therapeutic agents for the treatment of numerous medical disorders. Seven types of toxins have been harvested from clostridium, designated A through G, but only type A (BTX-A) and B (BTX-B) are commercially available and used in clinical practice. In 1989, the Food and Drug Administration approved BTX-A for the treatment of strabismus; since then, the growing use of this drug in several neurological disturbances has made it one of the most important advancements in the therapeutics of movement disorders such as muscular dystonia and dyskinesia. At the same time, botulinum toxin (BTX) either alone or adjunct to other measures has emerged as a new important therapeutic strategy for clinicians, treating a wide range of disturbances including gastroenterological and urological diseases as well as dermatological and cosmetic applications. 

In the last decade, one of the main indications of BTX is the treatment of disorders characterized by muscular hyperactivity and excessive or inappropriate muscle contraction. Neurorehabilitative medicine treats these patients and ameliorates severe neurological impairments that have scarcely available therapeutic interventions. This paper presents both the consolidated applications of BTX in spasticity as well as in other disturbances in which it has been shown to be a useful therapeutic tool. Since in clinical practice, BTX-B is less used than BTX-A, and few researches studies have been published regarding its use, most of data presented pertain to BTX-A treatment.

## 2. Structure and Type of BTX

The active BTX molecule is formed by two chains weighing ~150.000 daltons, in which a heavy chain is linked by a disulfide bond to a light chain [1]. The heavy chain is responsible for neuron-specific binding and internalization. Once internalized and within a vesicle, the light chain across the vesicle membrane is released into the neuronal cytoplasm, where it binds to a specific target protein involved in the docking and fusion of acetylcholine-containing vesicles to the internal portion of the cell membrane. These target proteins are collectively referred to as the SNARE complex. The BTX-A cleaves a protein termed SNAP-25, whereas BTX-B binds a different protein designed VAMP, also known as synaptobrevin [2]. Both are responsible for vesicle acetylcholine release. The derangement of this process at the neuromuscular junctions cause clinical effects consisting in muscle weakness and paralysis. BTX-A and BTX-B are commercially available and used in clinical practice. To date, three formulation of BTX-A are commercialized and are marketed as Botox (Allergan, Inc., Irvine, Calif, USA), Dysport (Ipsen Ltd., Berkshire, UK) and Xeomin (Merz, Frankfurt, Germany), respectively. The preparations are manufactured by different processes, have different formulations and potencies, which are determined by different biological assays based on their clinical use. BTX-B is marketed by Solstice Neuroscience (Malvern, Pa, USA) as MyoBloc in the United States and NeuroBloc (Elan Pharmaceuticals, San Diego Calif, USA) in Europe. It is important to note that the potency of a single unit varies greatly among the commercial types. Although the potency of 1 U of Botox is roughly equal to 1 U of Xeomin, 3 U of Dysport, and 40 to 50 U of MyoBloc, it is very important to recognize that a simple ratio of dosing equivalencies cannot be applied [3]. BTX-B is commercially packaged in vials of 10 mL containing 5.000 MU and 10.000 MU of neurotoxin. For muscle injections, botulinum toxins are diluted with 0.9% sodium chloride solution at variable volumes depending on the dose that the clinician plans to inject. BTX doses are generally adjusted according to factors such as severity of the hyperactive muscle, number of muscles involved, age, and previous response to BTX therapy. The duration of BTX effect is variable depending from several factors including type of neurotoxin, dose, site of injections, and clinical applications. In disease botulism, neurotoxin A produces longer paralysis than botulinum neurotoxin B consistent with human observations [4]. Likewise, BTX-A has been shown to have a longer duration of effect in cervical dystonia compared with BTX-B [5]. Botulinum types A and B have a similar duration of clinical action in treating drooling due to neurological diseases [6]. In poststroke spasticity, the duration of action was not specifically addressed by the available studies although some trials suggested that efficacy of BTX-A may be appreciated 6 weeks after injection and for up to 9–12 weeks [7]. BTX-B has a tendency to produce more autonomic side effects than BTX-A [8] and can have a more enduring action than BTX-A in specific clinical applications such as hyperidrosis and sialorrhea [9, 10].

## 3. Spasticity

In a neurorehabilitation setting, BTX is predominantly used for the treatment of spasticity and to prevent muscular contractures. Spasticity is defined as a velocity-dependent increased resistance to passive limb movement in people with upper motor neuron syndrome [11]. Clinically, it is an involuntary motor disorder, characterized by hypertonic muscle tone with increased excitability of the muscle stretch reflex and increased tendon reflexes. Muscle weakness and limb paresis are associated to spasticity and contribute to the loss of motor dexterity and ability. Spasticity, if left untreated can hamper functional outcome by promoting persistent abnormal posture that, in turn, produces muscular-tendon contractures and bone deformity. Secondary complications arising from spasticity include impaired movement, hygiene, self-care, poor self-esteem, body image, pain, and pressure ulcers. Furthermore, patients with severe spasticity can develop poor social participation and quality of life (QOL) [12]. Because of these clinical concerns and related high social costs [13], several therapeutic strategies have been proposed for the treatment of spasticity including surgical, medical, and rehabilitative procedures. However, spasticity is not always harmful and patients with a combination of muscle weakness and hypertonic muscles may rely on the increased tone to maintain their posture and aid standing or walking. It is important to point out that BTX use is indicated when the spasticity is focal or segmental and if it interferes with active or passive functioning. Treatment of muscle hyperactivity may be considered when the condition is disabling. The primary aim of the treatment of spastic muscles is to maintain length and allow normal positioning of the limbs to prevent secondary soft-tissue shortening. Generally, BTX treatment is carried out as an adjunct to other rehabilitative strategies that are based on an individualized, multidisciplinary programmes and targeted to achieve patient goals. Treatment plans must consider a tradeoff between reduction of spastic hypertonia and preservation of residual motor function [14]. Although there is no consensus as to when BTX therapy should be initiated, or how long it should last, BTX-A injection is considered the hallmark or first line of medical treatment for focal/segmental spasticity [15]. Conversely, BTX-B has been predominantly used as an alternative agent for patients who developed resistance to BTX-A [16]. BTX-B has been used for the treatment of adult and child spasticity, but its effectiveness is unclear [17]. Data in the literature are insufficient to recommend it for the treatment of children with spasticity [18]. It is known that spasticity can follow several neurological diseases such as stroke, acquired brain injury, multiple sclerosis, cerebral palsy, and spinal cord injury. BTX use for the treatment of spasticity in some of those will be addressed. 

### 3.1. Poststroke Spasticity

Spasticity is a frequent motor disorder in adult patients with stroke and its incidence is variable ranging from 17% to 43% [19–21]. A bulk of trials have demonstrated the efficacy of BTX-A in reducing poststroke spasticity [22–24]. Improvement of hypertonic muscles has been reported both in upper [12, 25–28] and lower limbs [29–31] after BTX-A injections. Thus far, administration modalities, and standard muscle doses of BTX-A (either Botox or Dysport) have been proposed for the reduction of limb spasticity in adult patients with stroke. However, there is no clear evidence from the literature to guide optimal timing of interventions (e.g., early versus late), frequency of interventions, dilutions, injection sites, or doses. Current clinical recommendations for muscle-specific dosing in spasticity remain largely based on expert opinion, clinical experience, as well as the formulation of botulinum toxin being used. A mean BTX-A global dose ranging from 90 to 360 MU and from 350 to 1500 MU per intramuscular injection has been reported for upper spastic limbs when using Botox or Dysport, respectively. A BTX-A dose ranging from 100 to 400 MU and from 400 to 1500 MU for the two toxins, respectively, has been used in treating lower spastic limbs [7, 32]. According to the European Consensus on the use of BTX-A in adult spasticity, maximum doses should not exceed 1500 MU Dysport and 600 MU Botox per injection session [33]. The magnitude of response is dose dependent [34, 35] even if the dosage is largely titrated by the practitioner based on individual patient response. 

BTX-B has also been used in treating poststroke spasticity, but its efficacy in reducing spasticity has been questioned and, thus far remains unclear [17]. A recent systematic review of BTX use in adult poststroke spasticity concluded that available data on BTX-B were insufficient to assess its effect on spasticity and that further controlled trials using BTX-B were necessary [7].

 Although the reduction of spasticity is widely demonstrated with BTX-A treatment, its impact on the improvement of dexterity and functional outcome remains controversial. Some functional improvements may be seen after BTX injections, but global functional assessment methods do not consistently reflect these changes. Numerous studies have reported to attain prespecified goals [26, 36], active movement [37, 38], and gait [39]. Conversely, other studies did not find any benefit on functional gain in patients with post-stroke spasticity after BTX-A treatment [12, 40–44]. Fridman et al. [45] reported kinematic parameters improvement of spastic upper arm in post-stroke patients after BTX-A injections. They speculated that the improvement in velocity and time required to perform some motor tasks could be translated to countless situations in a patient's life, which is difficult to determine with objective functional scales. Likewise, Bensmail et al. [44] described improvement of kinematic parameters in upper spastic arm after BTX-A treatment but without significant changes in clinical outcomes. However, clinical practice shows that some patients benefit with improved motor function, but that predicting factors still have to be identified. Several reasons have been suggested to explain this contrasting finding. It is possible that spasticity does not contribute to limitation of function and that the underlying weakness is the only significant cause of activity limitation [46]. Many recovering patients with stroke experience significant reductions in functioning, QOL, and family relationships. Improvements in QOL, caregiver burden, and patient functioning are key measures of success in any rehabilitation program. Furthermore, these patients can develop shoulder pain that interfere with the rehabilitative process and has been associated with poorer outcomes and prolonged hospital stays [47]. BTX-A treatment of poststroke spasticity has been demonstrated to produce improvement of patients' quality of life [12, 25] and pain relief [48].

### 3.2. Spastic Cerebral Palsy

Cerebral palsy (CP) is the most common nonprogressive cause of motor disturbance and disability in children. Even with improvements in medical technology and clinical practice, the overall rate of CP remains high, with 2 to 3 per 1000 live births [49]. CP is the main cause of spasticity in children. Therapeutic management may include splinting/casting, passive stretching, facilitation of posture and movement, spasticity-reducing medication, and surgery. Many clinicians frequently face the dilemma of whether and how to medically treat spasticity in children with CP. When pharmacologic intervention is deemed appropriate, treatment decisions must first be based on accurate assessment using valid and reliable clinical instruments, and, even more importantly, measurable, achievable, and realistic treatment goals should be delineated. Successful use of BTX in children with CP was first reported in 1993 by Koman et al. [50]. Since then, there has been a growing interest of the therapeutic effects of BTX-A, and many trials have investigated its effectiveness for treatment of spasticity in children and adults with CP [18, 51–55]. Likewise in poststroke spasticity, BTX-A has been effective in the reduction of spasticity of both upper [52, 56] and lower extremities [54, 57]. 

In the late 1990s, pediatric doses of BTX-A in the treatment of children with spastic CP ranged from 12 to 16 U/kg and from 15 to 25 U/Kg of body weight for Botox [58] and Dysport [59], respectively. These dosages have increased over time [60]. Higher BTX-A doses of 15 to 22 U/kg and of 20 to 30 U/kg have also been used without serious adverse events for Botox [61] and Dysport [62, 63], respectively. Maximum doses of BTX-A should not exceed 300 U Botox and 900 U Dysport per injection session, respectively. Children typically receive higher doses per kilogram of body weight than adults and can develop more adverse events. BTX-A treatment is effective and safe, maintaining long-lasting effects after repeated injections. A recent review of relevant literature concerning the treatment of spasticity in children with CP recommended that the use of BTX-A should be offered as an effective and generally safe treatment to reduce localized/segmental spasticity in upper and lower extremities. The same review did not find sufficient data to support or refute the use of BTX-B [18]. In this rehabilitation area, BTX-A is generally used as an adjunct to physiotherapy or other rehabilitative interventions such as casting or orthotics to obtain reduction of spasticity and functional improvement. A review by Ryll et al. [54] reported that BTX-A injections combined with usual care or physiotherapy can have a positive effect on walking in children with CP. Trials comparing BTX-A with usual care or physiotherapy showed evidence that functional outcomes improved at different follow-up times of 2 to 6 weeks [64, 65], 12 weeks [66], and 24 weeks [64, 66] when BTX-A injections were combined with usual care or physiotherapy compared to usual care or physiotherapy alone. Similarly, another recent systematic review found a high level of evidence supporting the use of BTX-A as an adjunct to managing upper limbs in children with spastic CP [56]. However, several issues remain unsolved including timing, duration of BTX-A action, and its effectiveness in the long term. Furthermore, when BTX-A is used as adjunct to other measures, the type of physiotherapy that is best indicated, application, timing, and type of casting to obtain better results remain unclear.

### 3.3. Brain Injury

About 75% of patients with physical disability following severe brain injury (BI) will develop spasticity requiring specific treatment. Also, patients with focal spasticity due to BI can benefit from BTX treatment. Unlike poststroke spasticity, scarce data about the use of BTX in treating spasticity following traumatic BI have been reported. Generally, these studies used BTX-A and enrolled small or heterogeneous samples of patients including subjects with stroke and traumatic BI [67–70]. BTX-A injection was used as an adjunct to physiotherapy [71] or casting [67] strategies. However, all studies demonstrated the efficacy of BTX-A in reducing spasticity either in adults [23, 67–70] or children [71, 72]. 

## 4. BTX as Adjunct Therapy for Management of Spasticity

Treatment of spasticity incorporating BTX is usually part of an integrated multidisciplinary rehabilitation program. BTX is rarely a sole treatment, and it is generally used combined with physiotherapy or casting, particularly in children with spastic CP. Physiotherapy procedures associated with BTX-A treatment can be variable including stretching posture, constraint induced therapy, occupational therapy, and electrical stimulation [73–76]. A previously mentioned systematic review reported that a combination of BTX-A and occupational therapy for the treatment of spastic arm in children with CP is more effective than occupational therapy alone in reducing impairment, improving activity level outcomes, and goal achievement [56]. Furthermore, the authors found a high level of evidence to support the use of BTX-A as an adjunct to managing upper limb spasticity in these children. 

### 4.1. BTX and Casting/Orthotics

BTX is also used to prevent muscular contraction and to facilitate cast application or orthotics. Serial casting is a method used for reducing contractures due to spasticity and can be applied both to upper and lower limbs. BTX can facilitate this process by producing temporary weakness and relaxation of the targeted muscles, allowing them to be stretched more easily, thus reducing the neurogenic and biomechanical components of spasticity. Casting is the application of fiberglass and/or plaster to the spastic upper or lower limb to immobilize a joint and has been proposed for the treatment of spasticity following stroke, acquired brain injury and CP, in particular. This strategy has been recommended as a treatment option in the management of equinus in children with CP for many decades. Early researches reported that BTX is as effective as serial casting in improving dynamic function for children suffering with cerebral palsy [77–79]. Hence, there has been a growing interest of the therapeutic effects of BTX-A as adjunct to casting [80–82]. However, the effect of BTX-A combined with casting on the reduction of spasticity remain controversial and unclear [53, 80–82]. A systematic review of the effects of the casting on equinus of children with CP did not find any differences between groups comparing BTX-A plus casting or BTX-A alone versus casting [81]. 

Furthermore, similar questions and doubts about the improvement of functional outcome were raised when BTX and casting was used for the treatment of spastic children with CP. Studies comparing BTX-A injections plus casting or BTX-A injections alone versus casting showed strong evidence for no effects on the functional outcomes in the application of BTX-A injections, casting alone, or a combination of both treatments [54] in children with CP. On the other hand, Yaşar et al. [82] recently observed that in chronic stroke patients, casting might be an appropriate intervention following BTX-A injection to prevent equinovarus deformity and to improve the quality of walking. Likewise, casting and application of orthotics might potentiate the effect of BTX treatment. Lai et al. [83] reported that in poststroke spastic patients, dynamic splinting after BTX injection increased the range of active elbow extension and suggested that it might be a useful adjunct procedure for optimizing BTX effects.

## 5. BTX Use without Spasticity

Neurological diseases can produce variable and complex impairments requiring tailored rehabilitation strategies. In a neurorehabilitation setting, clinicians have to approach numerous motor and nonmotor disorders other than spasticity. BTX use in specific neurological diseases and disorders with complex neurological dysfunction will be provided.

## 6. BTX and Focal Hand Dystonia

Focal hand dystonia (FHD) is a motor disturbance characterized by a task specific muscle spasms, in which learned or repetitive motor tasks (such as writing or playing a musical instrument) trigger muscle spasms and interfere with practiced motor execution, whereas other actions remain normal. FHD include a variety of disorders affecting many different skilled functions. Writer's cramp and musician's dystonia are the most common forms of idiopathic FHD [84]. Writer's cramp is characterized by involuntary, repetitive, or sustained contractions of finger, hand, or arm muscles that occur during writing and produce abnormal postures or movements that interfere with normal handwriting. Although the prevalence is relatively low, varying from 3 to 7/100 000, [85, 86], it may be responsible for considerable morbidity in terms of working impairment, pain, embarrassment, low self-esteem, and poor social interaction. Musician's dystonia is a task-specific movement disorder that manifests itself as a loss of voluntary motor control in extensively trained movements. Approximately 1% of all professional musicians develop musician's dystonia, and in many cases, the disorder terminates the careers of affected musicians [87]. Therapeutic strategy proposed for the treatment of FHD including muscle relaxation techniques, physical and occupational therapy, and medical and surgical therapies have all been disappointing. Several researches have demonstrated that BTX injections into selected hand and forearm muscles provides the most effective relief in patients with these task-specific occupational dystonias [88–90]. Injection of BTX into the muscles responsible for the abnormal postures can be very effective and is often considered the first choice. A muscle dose ranging from 5 to 40 MU of BTX-A (Botox) and from 15 to 150 MU of BTX-A (Dysport) has been injected for writer's cramp [91]. There has also been a specific study showing utility for musician's cramps [92]. Patients can continue to respond to injections for many years. Lungu et al. recently reported that BTX-A treatment was safe and effective after more than a decade of treatment [93] in 20 patients with FHD. Of these, the musicians were more likely to wait longer between injections. The dose of BTX is based on the size of the muscle affected, the intensity of the spasm, and the number of muscles involved [94]. Likewise, in other motor disorders, BTX can correct abnormal hand posture and relieve discomfort. However, the restoration of normal hand function can be difficult to achieve. Not only are some of the underlying dystonic defects, such as loss of speed and coordination, not fully addressed by the botulinum toxin injection, but the weakness that accompanies injection can be an additional source of hand disability. In some patients, the tradeoff between disability due to weakness from injection and disability due to the dystonia itself is not acceptable. The cyclic improvement with BTX injection and the worsening of the dystonia when the benefit wears off, does not allow for consistent sustained performance which is especially problematic for professional musicians [95]. 

## 7. BTX and Obstetrical Brachial Plexus Injury

Obstetric brachial plexus injury (OBPI) can be a dramatic sequela of dystocia or complicated delivery. A recent study showed an incidence of 1.3 per 1000 live births in the United States [96]. A higher incidence, ranging from 3 to 4.6 per 1000 live births was found in Europe [97, 98]. Severe brachial plexus palsies can result in disabling due to impairment and imbalance of the muscular contraction in the paretic limb. In spite of physical therapy, some children continue to experience contractures and abnormal posture that hamper complete recovery. In the last decade, an increasing number of reports on the treatment of BTX-A for OBPI have been published [99–103]. BTX-A has been used to improve muscular imbalance of the internal rotator-adductor muscles of the shoulder, limited active elbow extension, and triceps cocontraction in combination with conservative treatment, including long-term physiotherapy, occupational therapy, and functional orthopaedic or plastic surgery. Furthermore, BTX-A as adjunct to serial casting has been successfully used in children with OBPI to improve muscular contracture, arm position, elbow extension, and dexterity in the paretic limb [99, 100]. However, a recent systematic review about the treatment indications of BTX-A in children with OBPI emphasized the need for randomized controlled trials to determine its benefits and efficacy in order to support the continued use of this intervention in managing muscle imbalance and muscle cocontraction in children with OBPI [104].

## 8. BTX in Sialorrhea

Since BTX also inhibits the release of presynaptic acetylcholine at the neurosecretory junctions of the salivary glands, it has been proposed as a possible efficacious pharmacological treatment for hypersalivation and sialorrhea, which can occur and complicate the course and management of some severe neurological diseases such as amyotrophic lateral sclerosis (ALS), Parkinson's disease (PD), and CP. Indeed, numerous studies have demonstrated that BTX-A and BTX-B are effective and safe for the reduction of drooling in patients with ALS and PD [105–108]. The injected doses into salivary glands is variable depending on the disease, the BTX used, and the clinician's experience. The mean global doses injected into salivary glands ranged from 55 U to 200 U (Botox) [109, 110] and from 250 U to 450 U (Dysport) [111, 112] for BTX-A and from 2500 to 4000 U for BTX-B [106, 108, 113]. A recent research study comparing the two toxins in controlling sialorrhea of ALS and PD patients reported that either 250 U BTX-A (Dysport) or 2500 U BTX-B (Neurobloc) have similar effectiveness and safety [6].

In children with CP, drooling and sialorrhea have an incidence of 10% to 37% [114]. These symptoms can have a devastating effect on the family's social relationships and the patient's quality of life. Several studies have demonstrated that BTX-A can be used with success in controlling sialorrhea in children with CP [115, 116]. A mean dose of BTX-A (Botox) ranging from 2 to 22.5 U/kg of body weight per single gland has been injected [117–119]. BTX-B can also be a safe and effective therapy for the treatment of drooling in these children. A recent randomized trial comparing three doses of 1500 MU, 3000 MU, and 5000 MU BTX-B injection into the salivary glands with ultrasound guidance [10] reported that the 3000 MU injection of BTX-B significantly improved the frequency and severity of sialorrhea in those children. The lower dosage was ineffective, and the higher dosage produced no greater benefit and more side effects. It has been proposed that BTX-B is more effective and could have a more enduring effect than BTX-A on autonomic function [10, 106]. Indeed, autonomic side effects are sometimes observed far from the injection site (such as dry mouth) after treatment for axillary hyperhidrosis with BTX-B [120]. As previously mentioned, BTX-B has a tendency to produce more autonomic adverse events than BTX-A [5, 8, 121, 122], mainly due to the hypothesized affinity for postganglionic neurons containing M3 receptors (such as those responsible for salivation) [123]. However, the study of Guidubaldi et al. found that that BTX-B had a shorter latency than BTX-A and comparable duration [6]. The most relevant finding was that BTX-B had a significantly shorter latency than BTX-A (3 versus. 6 days). The different latencies might be due to various characteristics of the two serotypes, perhaps diffusion and/or affinity for autonomic fibers. 

## 9. BTX Use in Rare Rehabilitative Clinical Conditions

Neurologically disabled subjects can present with complex dysfunction and clinicians have to face unique and difficult to treat clinical conditions. BTX can be a useful therapeutic tool in some of these conditions. Anecdotal reports have been published concerning the use of BTX-A in specific rare conditions such as sustaining posture after surgery in patients with cervical disk herniation, secondary to dystonic cerebral palsy [124]. BTX-A has been used to hasten the healing of lower lip ulcers due to oromandibular dyskinesia in a subject in a vegetative state following a severe subarachnoid hemorrhage [125]. Likewise, BTX-A treatment was used to hasten the healing of a buttock pressure sore in a subject with severe spastic paraplegia following a traumatic spinal cord lesion. In this last case, several therapeutic agents were applied without success, since all efforts at healing the ulcer by topical medication were hampered by recurrent spasms involving the gluteal muscles and the ulcer region [126]. Gluteal injections of 660 U BTX-A (Dysport) reduced the movement disorder and improved buttock ulcer healing.

## 10. Adverse Events

Before performing BTX-A injections for therapeutic purposes, the expected risks and benefits for each patient must be carefully considered. Currently, dosages are largely titrated by the practitioner based on the previously mentioned criteria and the individual patient's response. Reported adverse events associated with BTX are infrequent and predominantly concern the BTX-A formulation. Local and remote effects following BTX injections have been described. The former consisted in a reaction at the injection site, including pain, rush, and edema, whereas remote effects are due to diffusion of toxin and cause variable effects characterized by autonomic, regional, or systemic muscular weakness. Most adverse events after BTX treatment arise through weakness of the muscles injected or those nearby, which become weak through local spread of the toxin. Allergic or possible immune-mediated mechanisms have been proposed to be the cause of symptoms such as general malaise, fever, and skin rush. 

Observed adverse events include nausea, urinary incontinence, falls, seizures, fever, dry mouth, and dysphagia [127]. These disturbances have often been found in patients with preexisting comorbidities, for example, seizures in subjects with previous epileptic disorders. General malaise and “flu-like” symptoms have also been described [42]. Generally, they are mild to moderate and transient. A pooled analysis including 792 patients concluded that nausea was the most frequent minor adverse event in poststroke patients treated with BTX-A, affecting 2.2% of cases [127]. No serious adverse event was reported in a recent systematic review regarding BTX-A use in adults with poststroke spasticity [7]. Conversely, because children receive higher doses per kilogram than adults, they can develop more adverse events. A variable incidence of side effects ranging from 4% to 7% has been reported [40, 128, 129]. In a previously mentioned paper, 28.5% of CP children who were injected with 5000 MU of BTX-B for sialorrhea developed generalized weakness and severe dysphagia requiring hospitalization and nasogastric tube feeding [10]. A very infrequent systemic effect manifested was generalized weakness distant from the site of injection [130]. A recent review of cases described in the literature indicates that risk of developing systemic effects does not seem to be related to dose based on body weight [131]. It may be more likely that risk for this condition is related to the total injection dose and injection frequency. Doses greater than 600 units of Botox with follow-up injections occurring every 3 months may lead to an increased risk of developing severe adverse events. Repeated contralateral weakness and fatigue after high doses of BTX-A injection for poststroke spasticity have been also described [132].

## 11. Neutralizing Antibodies

BTX-A effects can be abolished by the development of neutralizing antibodies (NAbs). Antibody formation against BTX proteins is one of the reasons for therapy failure. Studies have demonstrated that antibodies-binding toxins, specifically in the region responsible for entry into neurons, neutralize or inactivate the toxin. In order to overcome therapy failure, injecting increased BTX doses with short injection intervals and using different BTX serotypes [133, 134] has been suggested. This phenomenon is reported with a variable incidence according to the treated disorder. In cerebral palsy, the incidence of NAbs has been reported in a range from 6% to 31% [128, 135, 136]. NAbs are rare in poststroke spasticity. In a sample of 235 poststroke patients with spasticity receiving a dose ranging from 100 to 400 MU of BTX-A, Yablon et al. [137] found <0.5% of NAbs. The development of NAbs are facilitated if repeated injections and high dosages of BTX are used independently from the treated disorder. However, NAbs are more frequent in patients with cervical dystonia compared to other hyperactive muscular disturbances. The development of NAbs has been also observed in subjects who underwent BTX injections for nonmotor disorders such as sialorrhea. Although no BTX-A resistance in the treatment of sialorrhea has yet been reported, this disappointing phenomenon has recently been described for BTX-B after repeated injection into the salivary glands [138].

## 12. Considerations

Muscle selection is a key feature for the efficacy of BTX treatment, and the infiltration modalities are a further source of heterogeneity. BTX-A injections are more efficacious if the muscles are targeted by needle EMG or ultrasound guidance. There is evidence from dystonia that EMG targeting increases accuracy and improves outcome [139]. However, when high doses are injected into sufficiently large muscles, as in spasticity, toxin diffusion compensates for this limitation. Salivary glands are generally injected by ultrasound guidance. A drawback for BTX therapy is its high cost and the transient nature of the toxin. In this respect, recent papers have reported that the clinical benefits of BTX-A treatment outweigh the apparent high costs of this intervention, showing it to be a cost-effective treatment [13, 42].

## 13. Conclusions

Botulinum toxin types A and B are valuable agents in the multiple therapeutic strategies that clinicians carry out in a neurorehabilitation setting. It is important to strive to attain the best clinical and functional benefit that improves the quality of care of patients undergoing rehabilitation. Since neurologically disabled subjects present complex dysfunction, prior to initiating BTX therapy, specific functional limitations, goals, and expected outcomes of treatment should be discussed with the patient and caregiver. Muscle selection and the order and priority of treatment should be tailored to the treatment of spasticity and muscular imbalance. BTX-A and BTX-B strategies should be viewed as adjunct measures based on the individual's residual ability, and competence and tailored rehabilitation programs are needed to achieve the best functional outcome. Although BTX-A treatment has been demonstrated safe and effective in managing several neurological disorders, many questions still remain unsolved. Future studies should address if this agent alone or as an adjunct to other rehabilitative procedures optimizes functional outcome.
